# Prognostic value of prognostic nutritional index score and controlling nutritional status score in patients with glioblastoma: A comprehensive meta-analysis

**DOI:** 10.3389/fonc.2023.1117764

**Published:** 2023-02-16

**Authors:** Jie Peng, Xiaoyu Li, Mingsheng Huang, Mincai Ma, Qin Huang, Ning Huang, Yuan Cheng

**Affiliations:** ^1^ Department of Neurosurgery, The Second Affiliated Hospital of Chongqing Medical University, Chongqing, China; ^2^ Hepatology Department, Chongqing Hospital of Traditional Chinese Medicine, Guizhou University of Traditional Chinese Medicine, Chongqing, China

**Keywords:** controlling nutritional status score, prognostic nutritional index score, CONUT score, PNI score, glioblastoma, prognostic value

## Abstract

**Introduction:**

Several nutritional indicators, including the prognostic nutritional index (PNI) score and the controlling nutritional status (CONUT) score, have been shown to predict the prognosis of patients with glioblastoma. The present meta-analysis was performed to further evaluate the prognostic value of PNI and CONUT scores in patients with glioblastoma.

**Method:**

The PubMed, EMBASE and Web of Science databases were comprehensively searched for studies that evaluated the ability of PNI and CONUT scores to predict the prognosis of patients with glioblastoma. Hazard ratios (HR) and 95% confidence intervals (CIs) were calculated by univariate and multivariate analyses.

**Result:**

Ten articles were included in this meta-analysis, involving 1406 patients with glioblastoma. Univariate analyses showed that a high PNI score was predictive of greater overall survival (OS; HR 0.50; 95% CI, 0.43, 0.58; I^2 =^ 0%) and progression free survival (PFS; HR 0.63; 95% CI, 0.50, 0.79; I^2 =^ 0%), whereas a low CONUT score predictive of longer OS (HR 2.39; 95% CI, 1.77, 3.23; I^2 =^ 25%). Multivariate analyses showed that high PNI score (HR 0.64; 95% CI, 0.49, 0.84; I^2 =^ 24%) and low CONUT score (HR 2.79; 95% CI, 2.01, 3.89; I^2 =^ 39%) were independently associated with longer OS, whereas PNI score was not significantly associated with PFS (HR 1.02; 95% CI, 0.65, 1.59; I^2 =^ 0%).

**Conclusion:**

PNI scores and CONUT scores have prognostic value in patients with glioblastoma. Additional large-scale studies, however, are required to confirm these results.

## Introduction

Glioma is the most common primary malignant tumor of the central nervous system. The World Health Organization (WHO) has classified gliomas as Grade 1 to Grade 4 malignancies, with glioblastomas having wild-type isocitrate dehydrogenase (IDH) being classified as Grade 4 tumors (1, 2). Although the comprehensive treatment of glioma has significantly improved, its prognosis remains poor, with the 5-year survival rate in patients with glioblastoma being <5% (3). Treatment of glioblastoma may be further improved by identifying factors contributing to the early prediction of patient prognosis.

Several inflammatory indicators have been shown to predict the prognosis of patients with glioma, including platelet-to-lymphocyte ratio (PLR), lymphocyte-to-monocyte ratio (LMR), neutrophil-to-lymphocyte ratio (NLR), and the albumin to globular albumin ratio (AGR) (4–6). In addition, several nutritional indicators, including prognostic nutritional index (PNI) scores, which are based on serum albumin concentrations and total lymphocyte counts, and controlling nutritional status (CONUT) scores, which are based on serum albumin and total cholesterol concentrations and total lymphocyte counts, have been reported to be independent predictors of overall survival (OS) in patients with various tumors. PNI scores and CONUT scores reflect the nutritional, inflammatory, and immune status of patients and have been found to predict the prognosis of patients with gastrointestinal tumors, hematological malignancies, and tumors of the urinary and reproductive systems (7–11).

Recent studies have also indicated that PNI and CONUT scores are predictive of OS and progression free survival (PFS) in patients with glioma (5, 12–14). For example, OS was found to be longer in glioma patients with higher than lower PNI scores (5, 12–19). Lower serum albumin concentrations have been associated with increased nutritional risk and lower immune status, factors related to a reduced anti-tumor effect, in patients with malignant tumors (16, 20). Lymphocytes are essential components of the immune system involved in regulating immunity, with the cytotoxic activities of some classes of lymphocytes limiting tumor growth and metastasis and improving patient prognosis (21). Low serum cholesterol concentrations may be associated with low serum antioxidant reserves, thereby affecting tumor cell proliferation and immune responses (22–25). PNI score and CONUT score can simultaneously assess the nutritional status and immune status of patients. Thus, they may be better predictors of patient outcomes than individual indicators of inflammation or nutrition.

Although several meta-analyses have analyzed the prognostic roles of inflammatory and nutritional indicators in patients with glioblastoma, those analyses did not emphasize the prognostic value of PNI scores (6, 26). Several recent studies, not included in previous meta-analyses, have assessed the prognostic value of PNI scores in patients with glioblastoma (5, 13–16). Moreover, no meta-analyses to date have analyzed the prognostic role of CONUT score in patients with glioblastoma. The purpose of this study was to systematically review and further clarify the prognostic values of PNI and CONUT scores in patients with glioblastoma.

## Methods

This systematic review and meta-analysis conformed to the Preferred Reporting Items for Systematic Reviews and Meta-Analysis (PRISMA) guidelines (27).

### Literature search

The PubMed, EMBASE, and Web databases were comprehensively searched to identify studies published through November 23, 2022, assessing the prognostic values of PNI scores and CONUT scores in patients with glioblastoma. Search terms included “glioblastoma,” “malignant brain tumor,” “GBM,” “glial cell tumor,” “astrocytoma,” “glioblastoma multiforme,” “high grade glioma,” “prognostic nutritional index,” “PNI,” “controlling nutritional status score,” and “CONUT score.”

### Inclusion and exclusion criteria

The literature was searched independently by two authors (Peng and Li). Articles were included if (1) they included patients with glioblastoma (2); the endpoint was OS or PFS; and (3) they included CONUT and/or PNI scores that allowed the calculation of hazard ratios (HRs) and corresponding 95% confidence intervals (CIs). Studies by the same authors and those that had overlapping data were excluded, as were review articles, case reports, meeting summaries, letters, and preclinical (animal or cell) studies.

### Data extraction and quality assessment

The two authors (Peng and Li) independently selected relevant studies that met the inclusion criteria; any differences between these authors were resolved by discussion until a consensus was reached consensus. Information extracted from each study included author, year, country, patient age, gender distribution, outcomes and sample size (**Table 1**). The two authors (Peng and Li) independently evaluated the quality of each include article using the Newcastle Ottawa Quality Assessment Scale (4 points for quality of selection, 2 points for comparability, and 3 points for quality of results and adequacy of follow-up). High-quality studies were defined as those with scores ≥7.

**Table 1:** Summary of characteristics of the included studies.

Author	Year	Country	Age	Gender distribution	Outcome	Sample size
Xing-Wang Zhou	2016	China	median 53	W:34 M:50	OS	84
Wen-Zhe Xu	2017	China	mean 50.41	W:82 M:84	OS	166
Jin-Duo Ding	2018	China	mean 49.90	W:118 M:182	OS	300
Alessandra Marini	2020	Italy	NO Clear	W:59 M:65	OS、PFS	124
Chao Hu	2020	China	median 54	W:36 M:58	OS	94
Celine Garrett	2021	Australia	Median 63	W:33 M:54	OS、PFS	87
Hatice Yılmaz	2021	Turkey	Median 60	W:72 M:48	OS、PFS	120
Junhong Li	2022	China	Mean 53.41	W:91 M:185	OS	276
Qian He	2022	China	Median 50	W:43 M:48	OS	91
Ozkan Alan	2022	Turkey	median 52	W:19 M:45	OS	64

### Statistical analysis

All statistical analyses were performed using the Review Manager version 5.4 software. OS and PFS were reported as HRs and 95% CIs. LogHR and SE were calculated using the general inverse variance method. Combined HRs and 95% CIs were estimated using forest plots. When I^2^ was less than 50%, a fixed effects model is was; otherwise, a random effect models was used. Sources of heterogeneity were analyzed and evaluated by sensitivity or subgroup analysis. P-values <0.05 were defined as statistically significant.

## Results

**Figure 1:**
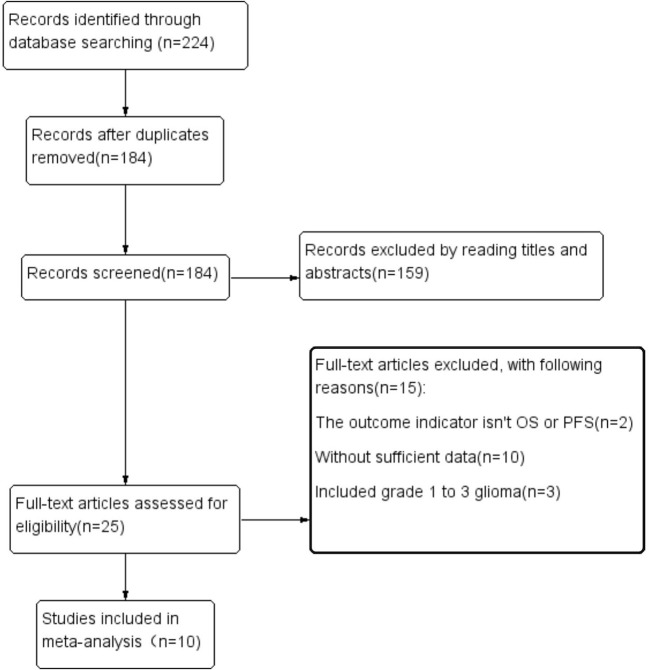
Flowchart of the article search performed.

The article retrieval process and results are shown in **Figure 1**. The initial search identified 224 articles; after deleting duplicate articles, reading titles and abstracts, and full text evaluation, 214 of these articles were excluded. Thus, ten studies were included, involving 1406 patients. All included articles had been published during the past 10 years and came from four different countries. Eight of these articles, involving 1036 patients, analyzed the predictive value of PNI scores, with all eight articles reporting OS and three reporting PFS. Three articles, involving 490 patients, analyzed the ability of CONUT scores to predict OS.

### Study characteristics and quality evaluation

**Table 1** summarizes the characteristics of the 10 included studies, including author, year, country, patient age, gender distribution, outcomes and sample size. Evaluation by Newcastle Ottawa Quality Assessment Scores showed that all 10 articles were of high quality, with five having 9 points and five having 8 points on this scale (**Table 2**).

**Table 2:** Quality evaluation of included studies using the Newcastle Ottawa Scale for cohort studies.

Author	Year	Selections	Comparability	Outcome	Score
Xing-Wang Zhou	2016	****	**	**	8
Wen-Zhe Xu	2017	****	**	**	8
Jin-Duo Ding	2018	****	**	***	9
Alessandra Marini	2020	****	**	***	9
Chao Hu	2020	****	**	**	8
Celine Garrett	2021	****	**	**	8
Hatice Yılmaz	2021	****	**	***	9
Junhong Li	2022	****	**	***	9
Qian He	2022	****	**	***	9
Ozkan Alan	2022	****	**	**	8

*: stands for one point in the quality scoring scale.

#### Impact of PNI score on OS

**Figure 2:**
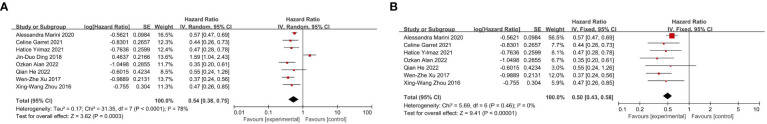
**(A)** Forest plot of correlation between PNI score and OS in univariate analysis data. **(B)** Adjusted forest map of correlation between PNI score and OS in univariate analysis data.

**Figure 3:**
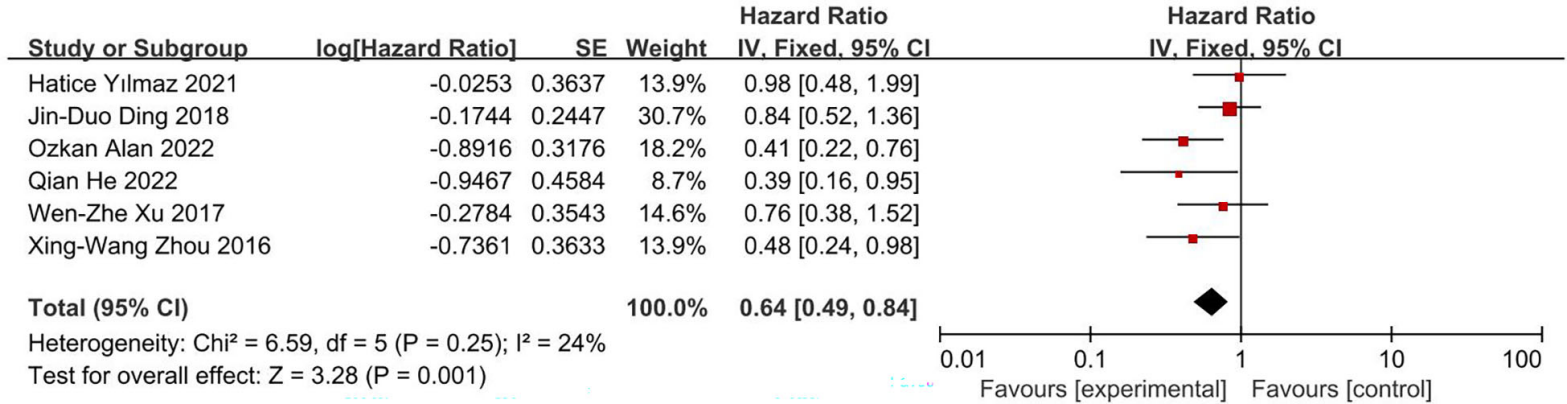
Forest plot of correlation between PNI score and OS in multivariate analysis data.

This analysis included eight studies, with all eight providing the results of univariate analysis and six providing the results of multivariate analysis. The pooled results indicated that a high PNI score was associated with longer OS in patients with glioblastoma. Due to the heterogeneity of these studies (I^2^ = 78%), a random effects model was used to process the results of univariate analysis, which found that the HR for high PNI score was 0.54 (95% CI, 0.38, 0.75; **Figure 2A**). However, after excluding one article (17), I^2^ decreased to 0%, resulting in an HR of 0.50 (95% CI, 0.43, 0.58; **Figure 2B**). The reason for the high heterogeneity is that in this article, there are differences between PIN scores of different ages and genders (17). Because the heterogeneity was not obvious (I^2^ = 24%), a fixed effects model was used for multivariate analysis data, resulting in an HR of 0.64 (95% CI, 0.49, 0.84; **Figure 3**). These results were all statistically significant.

#### Impact of PNI score on PFS

**Figure 4:**
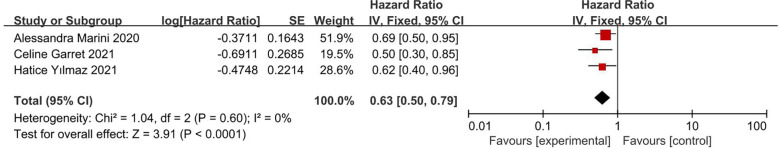
Forest plot of correlation between PNI score and PFS in univariate analysis data.

**Figure 5:**
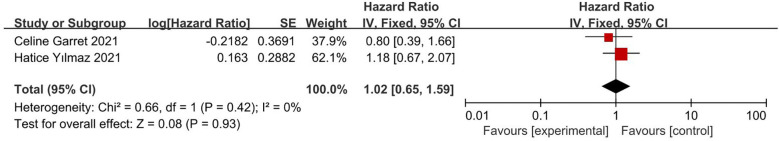
Forest plot of correlation between PNI score and PFS in multivariate analysis data.

This analysis included three studies, involving 322 patients, with all three provided the results of univariate analysis and two providing the results of multivariate analysis. The pooled results of univariate analyses showed that PNI score had an HR of 0.63 for PFS (95% CI, 0.50, 0.79; I^2 =^ 0%; **Figure 4**), whereas the pooled results of multivariate analyses showed that PNI score had an HR of 1.02 for PFS (95% CI, 0.65, 1.59; I^2 =^ 0%; **Figure 5**). The results of univariate analyses were statistically significant, whereas the results of multivariate analyses were not.

#### Impact of CONUT score on OS

**Figure 6:**
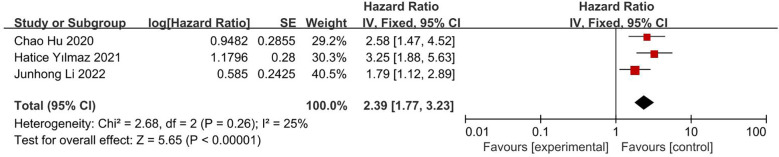
Forest plot of correlation between CONUT score and OS in univariate analysis data.

**Figure 7:**
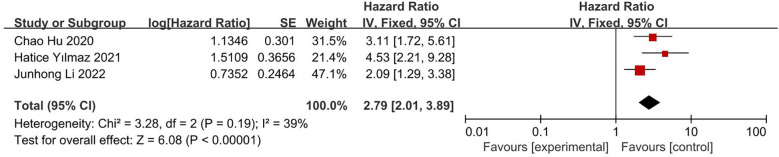
Forest plot of correlation between CONUT score and OS in multivariate analysis data.

This analysis included three articles, involving 490 patients. The results of both univariate analysis (HR 2.39; 95% CI, 1.77, 3.23; I^2^ = 25%; **Figure 6**) and multivariate analysis (HR 2.79; 95% CI, 2.01, 3.89; I^2^ = 39%; **Figure 7**) showed that low CONUT score was associated with significantly longer OS in patients with glioblastoma. Both I^2^ were less than 50% and statistically significant.

The number of studies that assessed the impact of CONUT score on PFS was insufficient for further analysis.

### Publication bias

Due to the limited number of studies included, the methods used to detect publication bias (such as funnel plots) would yield limited results. Therefore, publication bias was not evaluated.

## Discussion

In recent years, hematological factors were found to have predictive value in cancer patients, and meta analyses have been performed to evaluate the relationships between hematological indicators and clinical outcomes in these patients (28–32). The present meta-analysis therefore assessed the prognostic value of PNI scores and CONUT scores on outcomes in patients with glioblastoma.

Nutritional status, inflammatory status, and immune function have been associated with prognosis in patients with malignancy (15, 16, 23). PNI scores are based on total lymphocyte counts and serum albumin concentrations, whereas CONUT scores are based on these two factors and total cholesterol concentrations. Low serum albumin concentration is an indicator of poor nutritional status and activation of chronic inflammation. Activation of inflammatory responses releases various growth factors (GFs) and pro-inflammatory cytokines, such as interleukin-6 (IL-6), interleukin-1 (IL-1), and tumor necrosis factor-α (TNF-α) (20), altering catabolism in tumor patients. IL-6 stimulates the liver to produce C-reactive protein (CRP) and other acute-phase proteins, thereby increasing the demand for amino acids. Serum albumin and glutamine and serum albumin have been associated with chronic inflammatory responses to malignancy. Although most tissues can synthesize glutamine, the availability of this amino acid is limited during periods of rapid growth or stress. Glutamine demand is more pronounced in tumor cells, many of which exhibit an oncogenetic dependence on glutamine (33–35).

Lymphocytes are an important part of the immune system and are involved in immune regulation. The cytotoxic activity of lymphocytes limits the growth and metastasis of tumor cells. A decrease in lymphocyte counts may attenuate this effect (21, 36). Cholesterol is a major component of cell membranes and is associated with tumor cell proliferation and the immune response. Serum cholesterol concentrations were found to be lower in patients with malignant tumors, a reduction due to the proliferation of cancer cells (23, 24, 37). Low serum cholesterol concentrations have been associated with reduced levels of lymphocytes, total T cells, and CD8+ cells, impairing immune function (22, 23, 38). Hypocholesterolemia may be associated with low serum antioxidant reserves, which may increase susceptibility to oxidative stress (39).

The pooled results of the present meta-analysis suggested that PNI and CONUT scores are predictors of prognosis in patients with glioblastoma. Univariate analysis data showed that the relationship between PNI scores and OS was highly heterogeneous. Sensitivity analysis found that this high heterogeneity was caused by one of the included studies, with that study finding that the relationships between PNI scores and OS differed significantly by patient age and gender. Although age and gender were confounding factors in univariate analysis, their removal during multivariate analysis yielded an HR less than 1 (17).

This meta-analysis found had several limitations. First, although CONUT score was significantly prognostic value of OS, the number of studies and the patient sample size were relatively small. The small number of studies assessing the relationship between CONUT score and PFS precluded a meta-analysis of the relationship between these parameters. Additional studies with optimized designs are needed to validate these findings. Second, most of the included studies evaluated patients with glioblastoma, with relatively few studies evaluating patients with grade 3 glioma. Third, HRs and 95% CIs were calculated from univariate analyses data in the included studies, leading to an overestimation of the predictive effect. Fourth, most of the studies included in the meta-analysis were retrospective in design, as well as differing in cutoff values. Fifth, the extent of surgical resection may have affected the prognosis of patients with glioblastoma. Studies analyzing the effects of gross total resection (GTR), subtotal resection (STR) and biopsy on the prognosis of patients with glioblastoma have found that GTR is beneficial (13–16, 23). The studies included in the present meta-analysis did not group patients by surgical methods, precluding subgroup analysis to analyze the predictive effect of PNI and CONUT scores on the prognosis of patients with glioblastoma following different surgical methods. Sixth, the 2021 WHO classification of central nervous system tumors has categorized IDH mutant astrocytomas as Grades 2 to 4, IDH wild-type glioblastomas as Grade 4, and IDH mutant and 1q/19q deletion oligodendrogliomas as Grades 2 to 3. PNI and CONUT scores may have different prognostic effects in patients with IDH mutant and wild type tumors. Although mutant IDH has been associated with longer OS in patients with glioblastoma, the predictive effects of PNI and CONUT scores on patients with tumors carrying these two IDH genotypes were not determined separately, precluding a meta-analysis analyzing the predictive value of nutritional scores in patients with these genotypes. Finally, the small number of studies included prevented evaluation of the prognostic value of CONUT scores on PFS in patients with glioblastoma. Despite these limitations, however, the present study showed that both PNI and CONUT scores were valuable in determining prognosis in patients with glioblastoma.

## Conclusion

In conclusion, the present meta-analysis showed that both PNI scores and CONUT scores have prognostic value in patients with glioblastoma. These scores have several advantages, including their ease of determination and low cost. Additional large-scale studies, however, are required to validate these findings and determine the mechanisms by which nutritional status, systemic inflammation, and immune status affect prognosis in patients with glioblastoma.

## Author contributions

JP: conceptualization of the study; YC and JP: design of the study; JP, XL, QH, NH, MH, and MM: literature retrieval, study selection, statistical analyses, interpretation of the data and drafting of the initial manuscript; JP and XL: data extraction, quality evaluation; JP and YC: critical revision and comment for important intellectual content. All authors contributed to the article and approved the submitted version.
